# *Lactobacillus* (*Limosilactobacillus*) *reuteri*: a probiotic candidate to reduce neonatal diarrhea in calves

**DOI:** 10.3389/fmicb.2023.1266905

**Published:** 2023-10-03

**Authors:** Karin Schwaiger, Julia Storch, Christoph Bauer, Johann Bauer

**Affiliations:** ^1^Institute for Food Safety, Food Technology and Veterinary Public Health, Unit of Food Hygiene and Technology, University of Veterinary Medicine, Vienna, Austria; ^2^Veterinary Office Landratsamt Fürstenfeldbruck, Fürstenfeldbruck, Germany; ^3^Department of Quality Assurance and Analytics, Bavarian State Research Center for Agriculture, Freising, Germany; ^4^Chair of Animal Hygiene, School of Life Sciences, Technical University of Munich, Freising-Weihenstephan, Germany

**Keywords:** *Lactobacillus*, *Limosilactobacillus*, *reuteri*, calf, diarrhea, protection

## Abstract

**Background:**

Diarrhea in newborn calves is considered life-threatening and results in large economic losses in dairy farms. Lactobacilli generally play an important role in intestinal health, and *Lactobacillus* (*Limosilactobacillus*; *L.*) *reuteri* is the dominant *Lactobacillus* species in the feces of healthy calves during the first week of life. In calves with diarrhea on day 2 postpartum, lactobacilli are significantly reduced even up to 24 h before the onset of clinical signs. Since the probability of occurrence of diarrheal disease decreases as the *L. reuteri* count in the feces increases, oral administration of this species might have a protective effect against diarrhea.

**Objective:**

These studies were designed to demonstrate whether oral administration of preselected *L. reuteri* isolates can reduce the incidence of diarrhea in newborn calves on dairy farms.

**Microorganisms:**

46 *L. reuteri* isolates from 2-day-old healthy calves were available from a previous study.

**Animals:**

170 newborn calves of Simmental breed of 10 dairy farms in Bavaria (Germany), were included in the study; of 166 animals the data could be evaluated.

**Methods:**

Microbiological (antibiotic sensitivity test, acid and bile salt stability test, antimicrobial activity of the supernatants), molecular biological (PCR, RAPD-PCR) and toxicological methods (MTT test) were used to select and to characterize suitable *L. reuteri* isolates. The administration of a suspension of two selected *L. reuteri* isolates (6–8 × 10^8^ colony forming units per day) to calves was performed from day 2 to day 5 after birth in a double-blinded placebo-controlled study. Clinical monitoring of the calves continued until the 14th day of life.

**Results:**

Out of 46 *L. reuteri* isolates, only 2 met the set criteria and were used in the feeding trial. In the placebo group, 44 of 83 calves developed diarrhea within the first 2 weeks of life, whereas in the *L. reuteri* group this was only the case in 31 of 83 animals (*p* < 0.05).

**Conclusion:**

*L. reuteri* appears to be of particular importance for the intestinal health of newborn calves. The diarrhea protective effect could be even more pronounced if an improved administration regimen is developed in terms of start, frequency, and duration.

## 1. Introduction

Diarrhea in newborn calves is one of the most common multifactorial diseases in dairy farming. It is considered life-threatening and results in large economic losses in dairy farms (Elze et al., 1994; Doll et al., 1995).

The incidence of neonatal diarrhea varies from country to country. The lowest values are reported in Sweden and Denmark (9.8 and 10.3%) (Probo and Veronesi, 2022). In a former study carried out in Bavaria (Germany) we showed that about 37% of newborn calves developed diarrhea within the first 8 days of life (Schwaiger et al., 2022). Similar data were reported for Switzerland (Pipoz and Meylan, 2016), while incidence values from France (Lorino et al., 2005) and the Netherlands (Bartels et al., 2010) were lower (14.4 and 19.1%). Five to 10% of newborn calves die, and about half of the mortality in calves up to 1 month old is due to diarrhea (Kaske and Kunz, 2003; Brickell et al., 2009). The National Animal Health Monitoring System for U.S. dairy reported for 2014 that 56% of sick calves had digestive disorders with a mortality rate of 8.5%. Most cases of digestive disorders and death occurred in calves less than 1 month old (Urie et al., 2018).

The economic damage of neonatal diseases is not only based on the immediate events such as deaths, growth retardation and/or treatment costs, surviving animals mostly show negative effects on later health (e.g., increased animal losses until the end of the first lactation) and performance (e.g., later first calving age) (Donovan et al., 1998; Trilk and Münch, 2008).

Both infectious and non-infectious factors can cause diarrhea in calves. Known enteric pathogens include viruses like bovine rotavirus (BRV), bovine coronavirus (BCoV), bovine viral diarrhea virus (BVDV), bacteria like *Salmonella* (*S*.) *enterica*, *Escherichia* (*E*.) *coli*, *Clostridium* (*C*.) *perfringens*, and parasites like *Cryptosporidium* (*Cr*.) *parvum*. Additionally, emerging pathogens, e.g., bovine torovirus (BToV) or caliciviruses (bovine norovirus [BNoV] and Nebovirus) are known or suspected as causative agents of diarrheal disease (Cho and Yoon, 2014). Colostrum intake, housing type, and hygienic conditions are important non-infectious cofactors and may favor the development of clinical symptoms and influence the severity and outcome (Lee et al., 2019).

According to the current knowledge, the main prophylactic measures include comprehensive birth, housing, and feeding hygiene, timely and adequate colostrum supply to the calf, and vaccination of dams (Rademacher et al., 2011). Recently, we reported that *Lactobacillus* (*Limosilactobacillus*; *L.*) *reuteri* is the dominant *Lactobacillus* species in the feces of healthy calves during the first week of life (Schwaiger et al., 2020), which has since been confirmed (Fan et al., 2021). In addition, we showed that (i) in calves with diarrhea on day 2 postpartum, lactobacilli were significantly reduced even up to 24 h before the onset of clinical signs, and (ii) in particular, when the concentration of *L. reuteri* in the feces increased, the probability of occurrence of diarrheal disease decreased (Schwaiger et al., 2022). Several other studies described the probiotic effects of *L. reuteri*, e.g., vitamin and amino acid production, modulation of host immune responses and improvement of intestinal mucosa integrity (Aureli et al., 2011). This knowledge, together with the observations of our previous study, led us to hypothesize that possibly neonatal diarrhea in calves could be reduced by administration of *L. reuteri*, taking into account that *L. reuteri* have highly host specific effects (Duar et al., 2017). This paper describes the criteria for the isolation and selection of suitable *L. reuteri* strains explicitly from healthy newborn calves using clinical, microbiological, molecular biological and toxicological methods. This is followed by a description of an experiment on the effect of a mixture of two isolated *L. reuteri* strains on the incidence of diarrhea in calves during the first 2 weeks of life after oral administration.

## 2. Materials and methods

### 2.1. Origin of the *L. reuteri* isolates

In a recently published study (Schwaiger et al., 2020), we demonstrated that *L. reuteri* becomes the most common *Lactobacillus* species during the first week of life in healthy calves. A follow-up study has shown that lactobacilli were significantly underrepresented in calves with diarrhea on the second day of life, even 24 h before the onset of the disease. A total of 38 different species of lactobacilli were detected in the feces of all investigated calves (*n* = 150). A closer look at the *Lactobacillus* group using the logit model revealed the important role of *L. reuteri* in gut health, since the probability of diarrhea decreased significantly with increasing concentrations of *L. reuteri* (*p* = 0.036). Based on these results, we hypothesized that an earliest possible administration of *L. reuteri* might contribute to a reduced incidence of diarrhea (Schwaiger et al., 2022). A total of 46 strains of *L. reuteri* [identified by MALDI-TOF MS using a Bruker Microflex^™^ LT equipment (Bruker, 164 Billerica, USA) and the Biotyper Real Time Classification software v. 3.0 (Bruker Daltonics, Bremen, 165 Germany)] from the feces of 2-day-old calves (*n* = 38) of Simmental breed was available for the present study. *L. reuteri* DSM 20016 (German Collection of Microorganisms and Cell Cultures GmbH, Braunschweig, Germany) was included as a reference strain. Of these calves, *n* = 22 remained healthy during the first 14 days after birth, while *n* = 16 became ill with diarrhea. The calves were housed in calf-igloos or weather-protected individual boxes in 11 Bavarian dairy farms. All details on calf breeding regimes, data collection, as well as isolation and identification of bacteria can be seen in Schwaiger et al. (2020) and Schwaiger et al. (2022). Details on the breeding regimes of the present study are described below (see 2.5.1 Animals).

### 2.2. Characterization and selection of strains to be applied

#### 2.2.1. DNA extraction of the isolates

DNA was extracted from culture material of the 46 *L. reuteri* strains using the Power Soil^™^ DNA Isolation Kit (MoBio Laboratories Inc., USA) according to the manufacturer’s instructions.

#### 2.2.2. *L. reuteri* specific PCR

The primer pair REUT1 (5′-TGAATTGACGATGGATCACCAGTG-3′, forward) and LOWLAC (5′-CGACGACCATGAACCACCTGT-3′, reverse) synthesized by Metabion (Germany) was used to amplify a 1,000 bp sequence of the 16S rRNA gene (Chagnaud et al., 2001). The total volume of the PCR reaction was 25 μL, containing 12.5 μL of a commercially available master mix (PCR Master Mix, 2x, Promega Corporation, USA), to which 11.0 μL nuclease free water (Promega Corporation, USA), 0.25 μL (1 μM) primer REUT1, 0.25 μL (1 μM) primer LOWLAC, and 1.0 μL of template DNA were added. The reference strain *L. reuteri* DSM 20016 served as a positive control, nuclease-free water instead of template DNA as a negative control. The PCR was performed in a thermocycler (T3000, Biometra, Germany) under the following conditions: pre-incubation: 95°C, 600 s; amplification: denaturation 95°C, 30 s, annealing: 65°C, 30 s, elongation: 72°C, 60 s, 30 cycles; final elongation: 72°C, 600 s; cooling 4°C. The amplicons were separated by electrophoresis in a 1% agarose gel containing ethidium bromide and the profiles were visualized under UV-light.

#### 2.2.3. Randomly amplified polymorphic DNA-PCR (RAPD-PCR)

Isolates identified as *L. reuteri* were characterized by a RAPD-PCR as described by Williams et al. (1990) using the primer OPA-18 (5′-AGGTGACCGT-3′; synthesized by Metabion, Germany) (Mättö et al., 2004). The total volume of the PCR reaction was 25 μL, containing 12.5 μL of a commercially available master mix (PCR Master Mix, 2x, Promega Corporation, USA), to which 10.5 μL nuclease free water (Promega Corporation, USA), 1.0 μL (0.4 μM) primer OPA-18, and 1.0 μL of template DNA were added. The PCR was performed in a thermocycler (T3000, Biometra, Germany) under the following conditions: pre-incubation 95°C, 300 s; amplification: denaturation: 94°C, 60 s, annealing: 32°C, 1 s, elongation: 72°C, 120 s, 45 cycles; final elongation: 72°C, 600 s; cooling: 4°C. The amplicons were separated by electrophoresis in a 1% agarose gel containing ethidium bromide and the profiles were evaluated visually under UV-light.

#### 2.2.4. Determination of phenotypic antibiotic resistance

Antibiotic resistance of the *L. reuteri* isolates was determined by microdilution following the recommendations of the German Institute of Standardization DIN (DIN 58940-81: 2002) as previously described (Hölzel et al., 2010). Commercial microtiter plates pre-coated with various antibiotics in increasing concentrations (Merlin, Bornheim-Hersel, Germany) were used for this purpose. Briefly, a suspension of a *L. reuteri* strain in 0.9% NaCl solution was adjusted to Mc Farland 0.5 and 100 μL of this suspension were added to 13 mL Wilkins-Chalgren Broth (CM 643, Oxoid, Wesel, Germany). The wells of the microtiter plates were filled with 100 μL of the *L. reuteri* containing suspension, shaken for 5 min, sealed with an adhesive film, and incubated anaerobically [Anaerocult^®^ gas generator system (Merck, Darmstadt, Germany)] at 37°C for 48 h. The evaluation was carried out visually in consideration of the cut-off values specified by EFSA Panel on Additives and Products or Substances used in Animal Feed (2018): ampicillin: 2 mg/L; gentamicin: 8 mg/L; kanamycin: 64 mg/L; streptomycin: 64 mg/L; erythromycin: 1 mg/L; clindamycin: 4 mg/L; chloramphenicol: 4 mg/L.

Instead of tetracycline (cut-off value: 32 mg/L), we used doxycycline (cut-off value: 8 mg/L) for technical reasons (see Discussion for more details). Since *L. reuteri* is intrinsically resistant to vancomycin (Klein et al., 2000), testing according to EFSA guidance is not required. We first determined the sensitivity of isolates to clindamycin, doxycycline, erythromycin, gentamycin, and kanamycin. Isolates found to be sensitive to these antibiotics were then tested against ampicillin and chloramphenicol and then against streptomycin.

#### 2.2.5. Selection criteria for suitable isolates

The isolates eligible for further selection and characterization had to meet the following criteria in stages: (1) the species specific PCR must clearly confirm *L. reuteri*; (2) to avoid redundancies, *L. reuteri* isolates of the same sample and with the same RAPD profile were counted as one isolate; (3) the isolate must not originate from a calf with diarrhea; (4) the RAPD profile of an isolate from a healthy calf had to be found in at least 4 other isolates from 4 other healthy animals; and (5) the isolates had to meet the antibiotic sensitivity criteria.

### 2.3. Confirmation of identity of the selected strains by 16S rRNA-gene-PCR and DNA-sequencing

This PCR was performed on the finally selected *L. reuteri* isolates and the DSMZ reference strain and was carried out based on the method of Korthals et al. (2008) with the universal primer pair com1 (5′-CAGCAGCCGCGGTAATAC-3′, forward) and com2 (5′CCGTCAATTCCTTTGAGTTT-3′, reverse, synthesized by Metabion, Planegg, Germany) amplifying a fragment of approximately 400 bp of the 16S rRNA gene (Schwieger and Tebbe, 1998). The total volume of the PCR reaction was 25 μL, containing 20.37 μL nuclease free water (Promega Corporation, USA), to which 2.5 μL buffer (1.5 mM MgCl_2_), 0.5 μL dNTP mix (Quiagen, Germany), 0.25 μL (0.5 μM) primer com1, 0.25 μL primer com2, 0.13 μL hot start polymerase (Quiagen, Germany), and 1.0 μL of template DNA were added. The PCR was performed in a thermocycler (T3000, Biometra, Germany) under the following conditions: pre-incubation: 95°C, 900 s; amplification: denaturation 94°C, 60 s, annealing: 50°C, 60 s, elongation: 72°C, 70 s, 30 cycles; final elongation: 72°C, 300 s; cooling 4°C. The amplicons were separated by electrophoresis in a 1% agarose gel containing ethidium bromide and the profiles were visualized under UV-light.

Bands were excised from the gel with a scalpel and purified using a “Qiaquick Gel Extraction Kit” (Qiagen, Venlo, The Netherlands) as described by the manufacturer. Sequencing was carried out by Sequiserve (Vaterstetten, Germany), and the nucleotide sequences of the gene fragments were analyzed using the BLAST program.^1^

### 2.4. Further characterization of the selected isolates *L. reuteri* 6-1-5 Lac2 and 11-8-5 Lac 1

The *L. reuteri* isolates meeting the above-mentioned criteria were subjected to the following further investigations in order to confirm their general suitability for oral administration.

#### 2.4.1. Acid and bile salt stability

Since potentially probiotic strains have to survive the gastrointestinal passage, the acid and bile salt stability of the remaining *L. reuteri* isolates were tested based on the method of Saarela et al. (2003). The test parameters used were based on those of Vlková et al. (2009) and Maldonado et al. (2012) for the selection of probiotic bacteria for young ruminants. In brief: *L. reuteri* isolates were incubated anaerobically in PBS (pH 7.2), in PBS adjusted with 1 M HCl to pH 2 and 3, respectively, and in PBS containing 0.5 and 1.5% ox gall powder, respectively (Fluka, Germany) up to 4 h at 37°C under continuous shaking on a horizontal shaker (150 rpm, Titramax 1000, Heidolph, Schwabach, Germany). After 0, 2, 3 and 4 h, aliquots were taken and the germ concentration was determined by the reference spatula method on MRS-Agar (Oxoid, CM361, Germany) as described by Gedek (1974).

#### 2.4.2. Antimicrobial activity of supernatants of *L. reuteri* cultures against pathogens

An agar diffusion test as described by Schillinger and Lücke (1989) and modified by Maldonado et al. (2012) was used to investigate the antibacterial activity of supernatants of *L. reuteri.* Briefly, *L. reuteri* were incubated in MRS-bouillon (Merck, 1.10661, Darmstadt, Germany) for 18 h at 37°C under anaerobic conditions. The liquid culture was then centrifuged at 2,320 × *g* for 15 min. The supernatant was divided into two aliquots, the pH was determined, and one aliquot was adjusted to pH 6.5 using 1M NaOH. Before testing the antibacterial activity, the supernatants were filtered using a microfilter (0.2 μm, Sartorius, Göttingen, Germany). *E. coli* O101:K99 (Culture collection, Chair of Animal Hygiene, Technical University Munich, Freising-Weihenstephan, Germany), *S. typhimurium* (DSM 554) and *C. perfringens* (DSM 756) served as test organisms. The bacteria were suspended in physiological saline solution and the suspensions were adjusted to McFarland 0.5 (*E. coli*, *S. typhimurium*) and McFarland 2 (*C. perfringens*). Suspensions of *E. coli* and *S. typhimurium* were spread out to plate count agar (Oxoid, CM 271, Germany), those of *C. perfringens* to Schaedler agar (BD 212189, Becton Dickinson, Germany) containing 5% defibrinated sheep blood (1000100, Fiebig, Düsseldorf, Germany) and vitamin K_1_ (10 mg/L; 5.01890, Merck, Germany). Six holes (diameter 5 mm) were punched out of the agar using a sterile cork-borer (5 mm, VWR, Darmstadt, Germany) and filled with 35 μL of the native supernatants (pH 4.4) and with the supernatants adjusted to pH 6.5 by using NaOH. Pure MRS broth (pH 6.5) and pure MRS broth adjusted to pH 4.4 by using HCl served as controls. The test batches were left at room temperature (20–22°C) for 1 h to allow the liquid to diffuse into the agar. Subsequently, they were incubated aerobically or anaerobically (*C. perfringens*) at 37°C for 24 h.

#### 2.4.3. MTT-test

Even if *L. reuteri* is “generally recognized as safe” (GRAS; Food and Drug Administration [FDA], 2023), the presence of possibly cytotoxic compounds in culture supernatants was tested using the MTT- test (Mosmann, 1983). In brief: Culture supernatants were prepared as described by Motevaseli et al. (2013). Monolayers of Vero-cells cultured for 24 h in microtiter plate wells served as test organisms. Two different experiments were performed to exclude only acid-related effects: (1) pH values of an aliquot of the culture supernatants were corrected to 6.5 (equal to pure MRS broth) using NaOH; (2) pH of MRS broth was corrected to 4.3 (equal to culture supernatants) using lactic acid. Sterile filtered culture supernatant was added to the Vero cells in dilutions of 1:1 to 1:124 (diluent: RPMI 1640, R8658, Sigma, Germany) and incubated at 37°C for 24 h. Twenty-five μl of an MTT solution (3-(4,5-dimethylthiaol-2-yl)-2,5-diphenyltetrazolium bromide; 5 mg/mL phosphate-buffered saline) was added, incubated again for 1 h and then the optical density (OD) was determined photometrically at 490 nm. Addition of culture medium instead of culture supernatants to the Vero cells served as cell control. The relative cleavage activity (CA) was calculated using the formula.

$CA\;\left(\%\right)={OD}_{\mathrm{sample}}\times 100:{OD}_{\mathrm{cell}\;\mathrm{control}}$

### 2.5. Examination of the influence of the selected *L. reuteri* strains 6-15-5 Lac2 and 11-8-5 Lac1 on the incidence of diarrhea in newborn calves

#### 2.5.1. Animals

170 newborn calves of Simmental breed of 10 dairy farms (17 calves per farm) in Bavaria, were included in the study. Four calves developed diarrhea before the first administration of the *L. reuteri* suspension (or the placebo) and were therefore excluded from further evaluation. The farms had comparable calf management and feeding designs. In brief: All calves were placed outside within 24 h after birth in weather-protected individual boxes. Colostrum (fresh, hand-milked) was fed as soon as possible after birth. For the first 5 days of life and 3 to 4 times per day, the calves received hand-milked milk from the mother cow, followed by a milk replacer. During the perinatal period no antibiotics were administered to the cows. Further detailed information regarding management systems and data collection were reported earlier (Schwaiger et al., 2022).

To avoid data bias, no probiotic containing starter was fed. If necessary, oral electrolytes were the only symptomatic therapeutic agents when diarrhea occurred. Clinical examination of all calves was performed by the farmers after prior instruction for at least 14 days, and the results considering common clinical parameters, sensorium, sucking reflex, skin turgor, eyeball position, heart rate, respiratory rate, appetite, and feces characteristics, were recorded (Stöber, 2012). The scoring system of the Clinic for Ruminants of the Veterinary Faculty of the University of Munich (Germany) was used for the diagnosis: “feces that have the consistency of water or pea soup or that flow through spread fingers are classified as diarrhea” (Prof. Wolfgang Klee, personal communication).

#### 2.5.2. Preparation of *L. reuteri* suspension

The two selected *L. reuteri* strains were cultured in batches in 200 mL MRS broth for 24 h at 37°C under anaerobic conditions and constant shaking (150 rpm). The bacterial suspensions were centrifuged (370 × *g*), the pellets were washed three times with physiological saline, resuspended with buffered physiological saline (7.65 g NaCl, 0.724 g Na_2_HPO_4_, 0.21 g KH_2_PO_4_, distilled water ad 1,000 mL), and suspensions were photometrically adjusted to an extinction of 2.4 at *λ* = 600 nm (equivalent to 6–8 × 10^8^ cfu (colony forming units)/ml). In addition, the total *L. reuteri* count was checked by plating a dilution series on MRS agar (anaerobic, 48 h, 37°C). Equal amounts of the suspensions of the two test strains were combined and 10 mL were filled into sterile glass vials and sealed with autoclaved rubber stoppers under aseptic conditions. Purity of the prepared suspensions was controlled by cultural examinations. For this, 0.1 mL of the prepared suspension was plated in duplicate on plate count agar (Oxoid, CM 271), blood agar (plate count agar with 7.5% defibrinated sheep blood [Fiebig, 1000100]) and Gassner agar (Merck, 1.01282), and incubated for 24 h at 37°C. In addition, contamination with *Salmonella* was ruled out by selective enrichment. In brief: 1.0 mL of the *L. reuteri* suspension was added to 5.0 mL buffered peptone water (10 g peptone [Merck, 1.07213, Darmstadt, Germany], 5.0 g NaCl, 3.75 g Na_2_HPO_4_, 1.5 g KH_2_PO_4_, sterile distilled water ad 1000 mL) and incubated aerobically for 24 h at 37°C; then, 0.1 mL of the enrichment was dropped onto MSRV-agar (Oxoid CM 910, Oxoid SR 161) and incubated aerobically for 24 h at 37°C. *Salmonella* growth is characterized by a turbid, milky layer covering the previously clear agar.

#### 2.5.3. Placebo preparation and sterility control

Autoclaved corn starch (10 g) was suspended in 990 mL of buffered saline solution (7.65 g NaCl, 0.724 g Na_2_HPO_4_, 0.21 g KH_2_PO_4_, sterile distilled water ad 1,000 mL), and 10 mL of the suspension were transferred to sterile glass vials sealed with autoclaved rubber stoppers. Sterility of the placebo was checked by plating out 0.1 mL of each suspension on plate count agar, blood agar, Gassner agar and MRS agar and incubated for 24 h at 37°C under aerobic (all agar types) and anaerobic (blood agar) conditions. In addition, *Salmonella* enrichment was carried out as described above.

#### 2.5.4. Experimental design

In agreement with the Government of Upper Bavaria, the feeding experiment did not count as animal testing in the sense of the Animal Welfare Act due to the GRAS status of *L. reuteri*. However, a special permit was issued by Government in accordance with § 68, 2 of the Food and Feed Code (file reference: 56-2660-2416-13). Ten farms participated in the study, each with 15 to 17 newborn calves, with approximately the same number of animals per farm receiving *L. reuteri* suspension or placebo. A dose of 6–8 × 10^9^ CFU of *L. reuteri* or the placebo was administered orally to 83 calves each about 24 h after birth and on each of the following 3 days before morning feed. For the first 4 days, the calves received milk from the suckler cow, after which they switched to the usual milk replacer of the farm. The experiment was carried out as a double-blinded study: the farmers who cared for the animals and diagnosed “diarrhea” did not know whether they administered the suspension of *L. reuteri* or a placebo.

#### 2.5.5. Detection of pathogens by sandwich ELISA

In case of diarrhea during the first week after birth, the presence of the most prevalent diarrhea pathogens BRV, BCoV, *E. coli* F5 (K99), and *Cr. parvum* was tested by using a digestive antigen sandwich enzyme-immunoassay (ELISA; BIO K 348, Bio-X Diagnostics, Rochefort, Belgium) according to the manufacturer’s instructions.

#### 2.5.6. Statistics

All statistical analyses were carried out with the program “R” (version 4.0.3). For the cluster analysis dist(B, method “manhattan”), hclust(*, complete) was used. A generalized logistic mixed model (penalized quasi likelihood) that takes random effects into account was used to assess the differences between the *L. reuteri* and placebo groups (Bolker et al., 2009).

## 3. Results

### 3.1. Selection of *L. reuteri* isolates

#### 3.1.1. Species specific PCR

Species-specific PCR of the 46 *L. reuteri* isolates previously identified by MALDI-TOF-MS (Schwaiger et al., 2022) showed amplicons with a specific band at 1000 bp in gel electrophoresis. In 4 isolates, one or two additional bands were detected at 100 bp and 400 bp, respectively. These isolates were excluded from further investigations because a clear identification could not be guaranteed.

#### 3.1.2. RAPD-PCR

**Figure 1:**
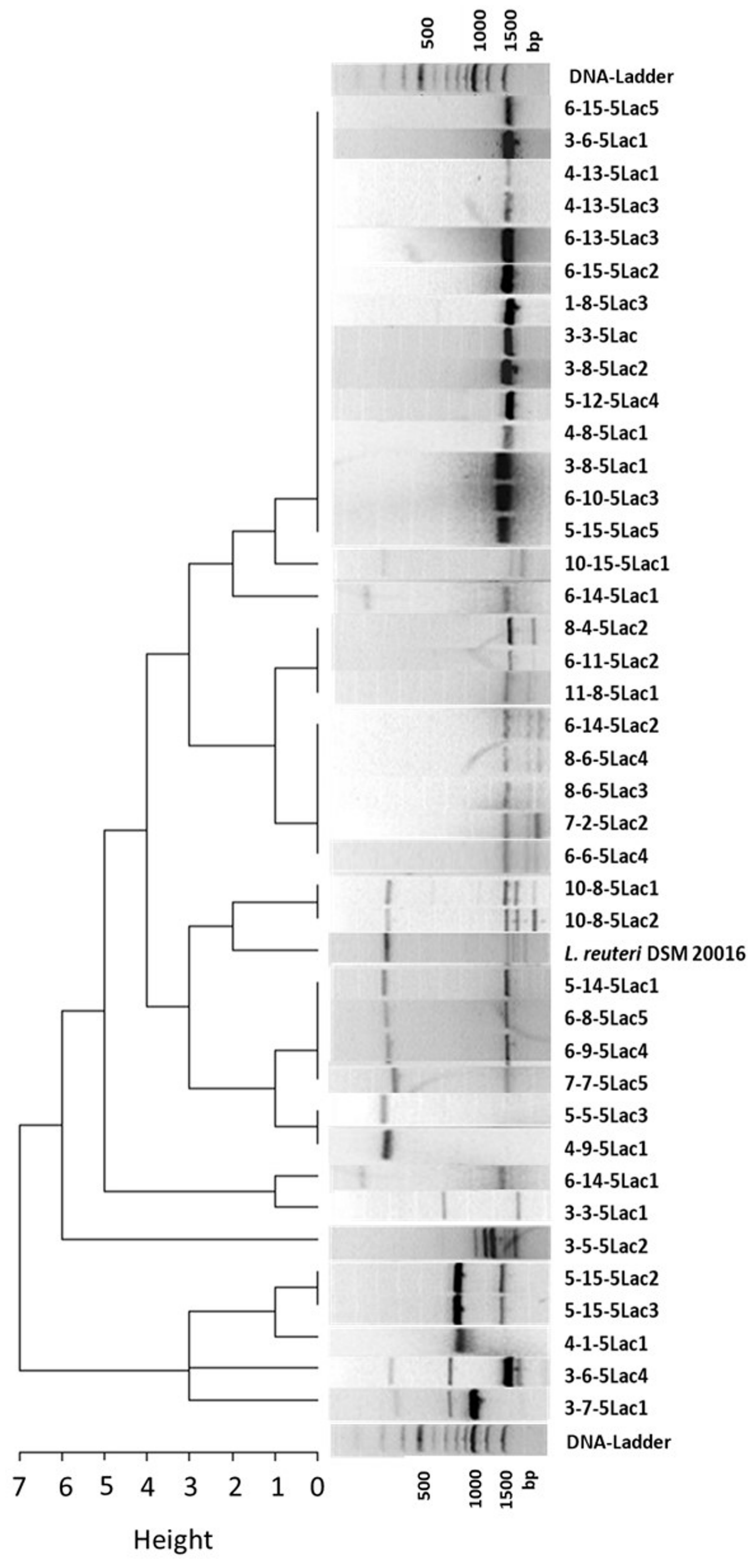
Cluster dendrogram of 40 *L. reuteri* isolates from healthy and diarrheic calves and the reference strain DSM 20016 based on their RAPD profiles (measured as Manhattan distances).

The remaining 42 isolates of *L. reuteri* and the reference strain DSM 20016 were characterized in more detail by RAPD-PCR. No amplicons were obtained from two isolates, so a total of 40 RAPD-PCR profiles were subjected to cluster analysis. In total, we were able to analyze 16 different profiles (9 single profiles and 7 profile-types derived from more than one isolate). The largest cluster comprises 13 isolates and is characterized by a single band at 1500 bp. The other clusters are formed by 2 to 5 isolates and usually have 2 or more bands. Details can be found in Figure 1.

**Figure 2:**
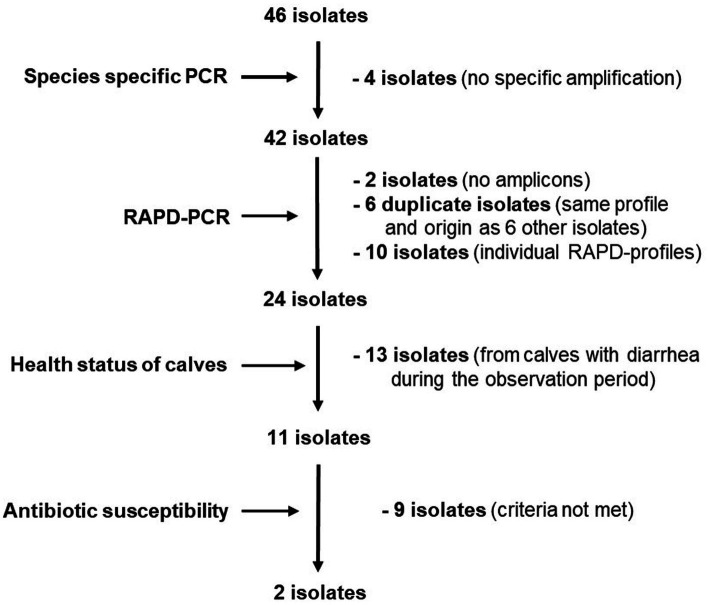
Flow chart depicting selection criteria and decisions for choosing potentially diarrhea protective *L. reuteri* isolates.

Considering the origin of the isolates, 6 isolate pairs with identical profiles originated from the same sample. Assuming that *L. reuteri* isolates with the same RAPD profile deriving from the same fecal sample are identical, the number of possible candidates was reduced to 34. Individual isolates with different RAPD profiles (*n* = 10) and isolates from animals that developed diarrhea within the observation period (*n* = 13) were not considered further. Since the probability of a possible protective effect might increase with the abundance of the strains in the feces of healthy calves, isolates from different calves with the most frequently shared RAPD profiles were selected for further investigations. The remaining 11 isolates were then subjected to susceptibility testing to selected antibiotics. Figure 2 summarizes the individual selection steps.

#### 3.1.3. Antibiotic resistance

In a first step, we tested the 11 isolates against clindamycin, doxycycline, erythromycin, gentamicin and kanamycin. Seven isolates did not meet the requirements. This was primarily due to resistance to clindamycin, erythromycin and/or gentamicin. All 4 remaining isolates were sensitive to chloramphenicol, but only 2 were sensitive to ampicillin. These two isolates (*L. reuteri* 6-15-5 Lac2 and 11-8-5 Lac1) were also susceptible to streptomycin. The results of the antibiotic susceptibility study of the *L. reuteri* isolates are summarized in Table 1.

**Table 1:** Minimum inhibitory concentrations (mg/L) of *L. reuteri* isolates in the microdilution test.

*L. reuteri* (isolate code)	Clindamycin (4)^1^	Doxycycline (8)^2^	Erythromycin (1)^1^	Gentamycin (8)^1^	Kanamycin (64)^1^	Ampicillin (2)^1^	Chloramphenicol (4)^1^	Streptomycin (64)^1^
81-8-5Lac3	1	**>16** ^3^	**2**	4	32	na^4^	na	na
3-3-5Lac3	0.5	**16**	**2**	**32**	>32	na	na	na
3-6-5Lac1	<0.063	2	0.5	2	15	**8**	4	na
6-6-5Lac4	0.125	4	0.5	1	32	**4**	4	na
6-10-5Lac3	**>8**	**16**	1	2	32	na	na	na
6-11-5Lac2	0.125	**16**	**2**	**16**	>32	a	na	na
6-14-5Lac2	**>8**	8	1	8	>32	na	na	na
6-15-5Lac2	<0.063	8	0.5	1	32	1	2	<8
7-7-5Lac1	**>8**	4	0.5	4	32	na	na	na
8-4-5Lac2	**>8**	4	0.25	2	>32	na	na	na
11-8-5Lac1	<0.063	4	0.5	2	16	2	2	8

^1^Cut-off value according to EFSA guidelines (2018); ^2^Estimated cut-off value for doxycycline [based on data of EFSA Panel on Additives and Products or Substances used in Animal Feed (2018) and Dallas et al. (2013)]; ^3^Reasons for exclusion are marked with bold letters; ^4^na: not analyzed.

### 3.2. Further characterization of *L. reuteri* 6-15-5 Lac2 and 11-8-5 Lac1

#### 3.2.1. Acid and bile salt stability

**Figure 3:**
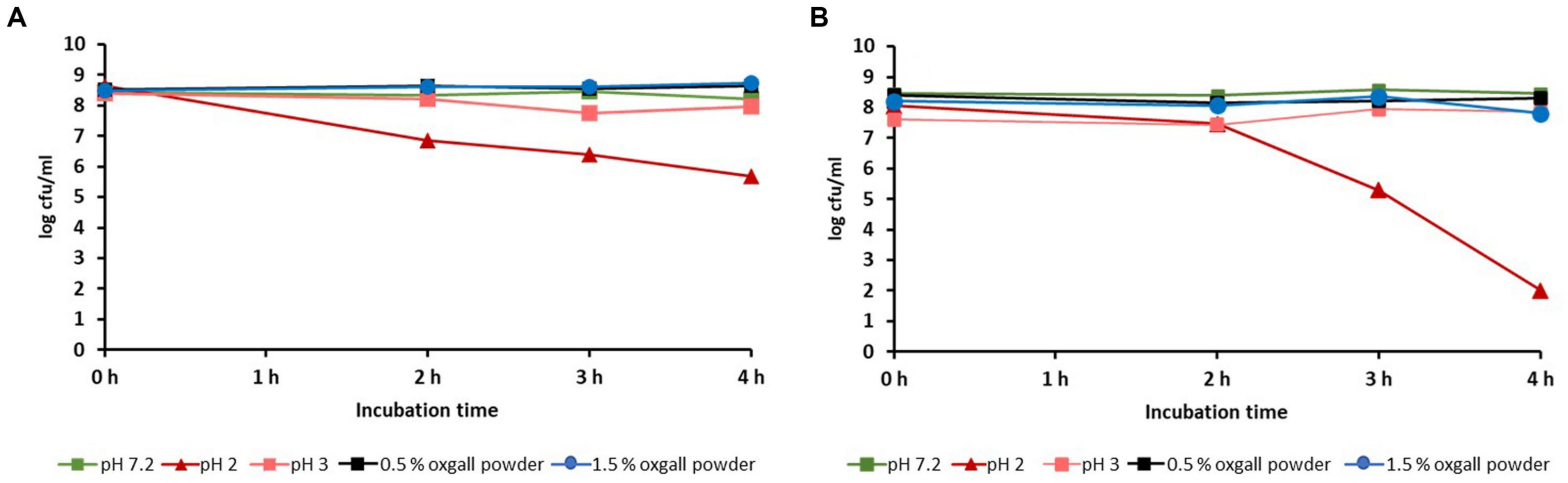
Influence of different pH-levels or oxgall powder concentrations on the viability of *L. reuteri* isolates 6-15-5-Lac2 **(A)** and 11-8-5 Lac1 **(B)**.

Both isolates were stable to a 4 h exposure to 1.5% bile salts or to pH 7.0 and 3.0; a decrease in the initial germ content (about 10^8^ cfu/mL) could not be determined. However, incubation of the isolates at pH 2 reduced the bacterial counts of *L. reuteri* 6-15-5 Lac2 and *L. reuteri* 11-8-5 Lac1 to 10^6^ cfu/mL and 10^2^ cfu/mL, respectively (Figure 3).

#### 3.2.2. Cytotoxicity of the supernatants determined by MTT test

**Figure 4:**
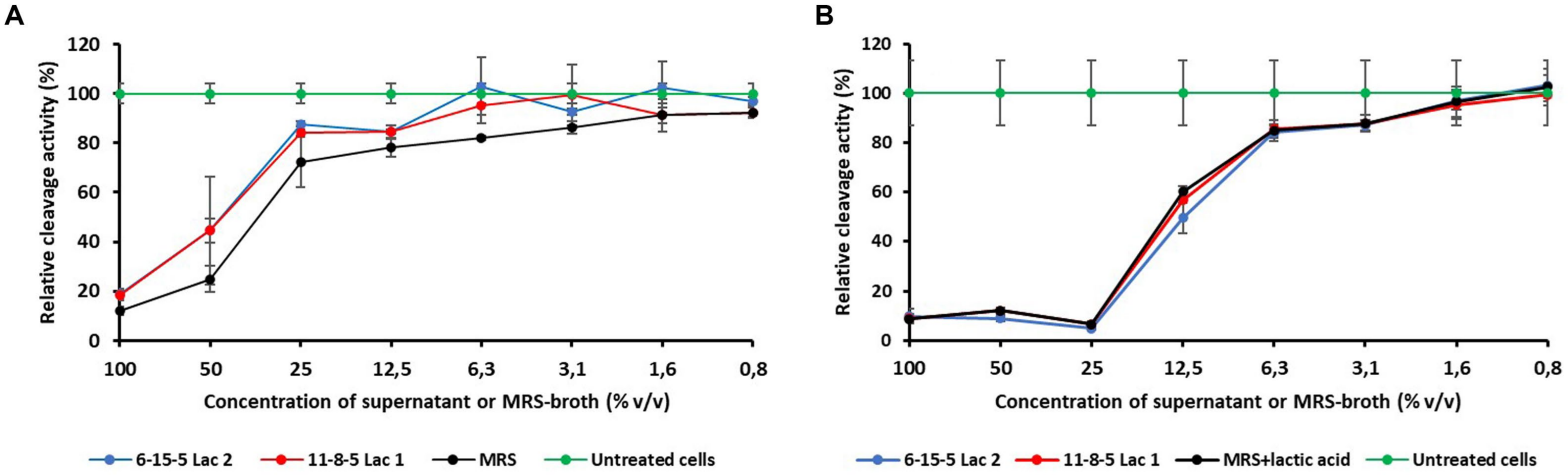
Influence of different concentrations of supernatans of *L. reuteri* isolates or MRS broth on the relative cleavage activity of Vero cells. **(A)** pH-values of the supernatants corrected to 6.5. **(B)** pH-values of the MRS broth corrected to 4.3.

During growth of the *L. reuteri* isolates in MRS broth, the pH changed from originally 6.5 to 4.3. To exclude only acid-related effects, two experiments with different pH values were performed (see Materials and Methods). The results are shown in Figure 4 and indicate that a pH level of 4.3 affects MTT cleavage activity more than pH 6.5. However, because the concentration-dependent courses of the cleavage activities were either nearly identical (pH 4.3) or even showed slightly higher values than the MRS control (pH 6.5), there is no evidence of acute cytotoxic activity in the supernatants.

#### 3.2.3. Antimicrobial activity of the supernatants

As already described, the growth of *L. reuteri* alters the pH of the culture medium. For this reason, the tests were carried out with and without pH correction. As depicted in Table 2, minimal inhibition of *E. coli* (K99), *S. typhimurium* (DSM 554) or *Cl. perfringens* (DSM 756) was recorded only by the native supernatant fluids of *L. reuteri* 6-15-5 Lac2 and 11-8-5 Lac1 (pH 4.4). On the contrary, neither pure MRS broth corrected to pH 4.4 nor culture supernatants adjusted to pH 6.5 formed inhibition zones.

**Table 2:** Inhibition of pathogens by supernatants of *L. reuteri* isolates (grown in MRS-broth) in an agar diffusion test.

Pathogen	Inhibition zone (mm)					
	6-15-5 Lac 2		11-8-5 Lac 1		MRS-Broth	
	pH 4.4	pH 6.5	pH 4.4	pH 6.5	pH 4.4	pH 6.5
*E. coli* O101:K99	3	0	4	0	0	0
*S. typhimurium* DSM 554	3.5	0	2	0	0	0
*C. perfringens* DSM 756	1	0	1	0	0	0

#### 3.2.4. DNA-sequences of the isolates *L. reuteri* 6-15-5 Lac2 and 11-8-5 Lac1

Analysis of the 2 isolates by species-specific PCR and 16S rRNA-gene-PCR revealed amplicons of approximately 1,000 bp and 400 bp, respectively. Analysis of the sequencing results by BLAST showed 99% sequence similarity for all PCR products (sequence length 409 and 999 bases) with the *L. reuteri* species of the database and between the two isolates. The sequences can be seen in Supplementary Table.

### 3.3. Influence of *L. reuteri* 6-15-5 Lac2 and 11-8-5 Lac 1 on the incidence of diarrhea in newborn calves

Of the 170 calves included in the study, 4 animals developed diarrhea within the first 24 h of life. Since the administration of the *L. reuteri* suspension or placebo only started on the 2nd day after birth, these animals were not included in the evaluation of the data, thus reducing the number of animals to *n* = 166.

The administration of the *L. reuteri* suspension or the placebo did not cause any immediate clinical abnormalities in any of the calves, neither in general behavior nor in feed intake.

**Figure 5:**
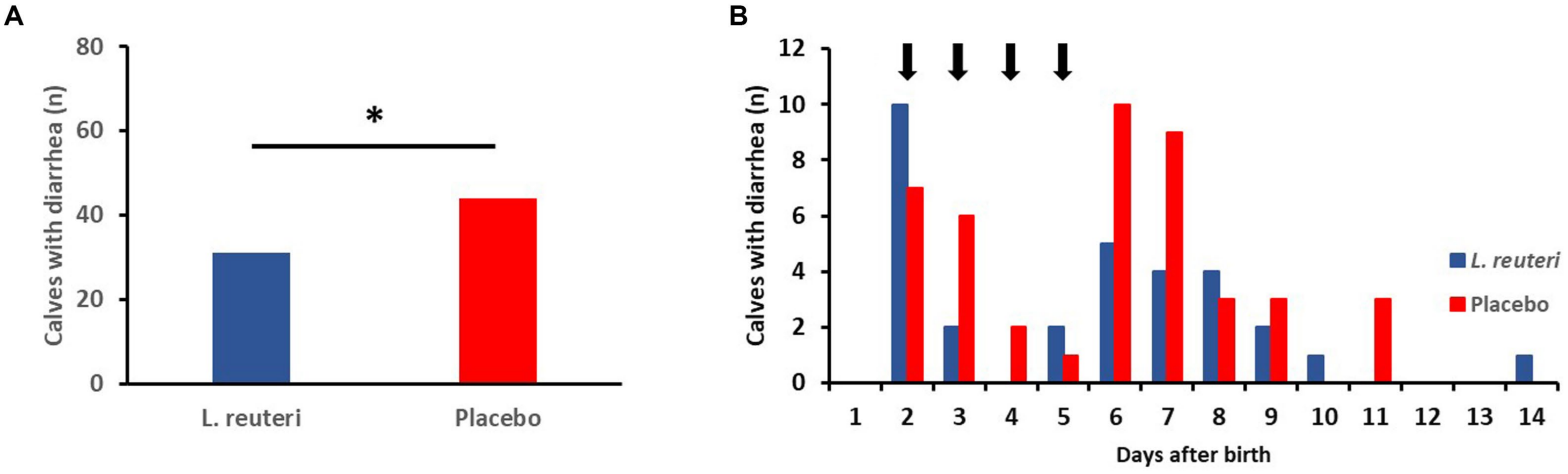
Influence of the administration of a combined suspension of *L. reuteri* isolates (6-15-5 Lac2, 11-8-5Lac1; 6-8×10^9^ cfu/dosis, once a day) or a placebo on the incidence of diarrhea in newborn calves (*n* = 83 per group). **(A)** Number of calves with diarrhea during the first 2 weeks after birth (*p < 0.05). **(B)** Time distribution of the onset of diarrhea (arrows indicate the administration of the suspension of *L. reuteri*).

Of the 166 calves included in the evaluation, 75 became ill with diarrhea in the first 2 weeks of life. Of these, 31 animals were in the *L. reuteri* group and 44 in the placebo group (Figure 5A); the difference is statistically significant (*p* = 0.048). Looking at the temporal course of the diarrhea incidences, it is noticeable that this difference is more pronounced between the 3rd and 10th day after birth (Figure 5B).

ELISA analysis of the diarrhea samples from the first week after birth for dominant pathogens showed that rotavirus and cryptosporidia were detected in 3 (13%) and 9 (39%) of 23 calves of the *L. reuteri* group, respectively, while this was the case in 10 (28%) and 15 (43%) of 35 calves of the placebo group. Coronaviruses were analyzed in 2 samples of the placebo group, whereas *E. coli* F5 (K99) was not detected in any sample. Overall, there were no significant differences in pathogen detection between the experimental and placebo groups.

## 4. Discussion

The effectiveness of the use of probiotics in calf husbandry in terms of increased performance and improved productivity parameters is well documented, but the available data on improved animal health and disease prevention seem to be less convincing (Alawneh et al., 2020). One reason for this observation may be that the probiotic strains used were not selected in a goal-oriented manner. To select a potentially diarrhea-preventive bacterial strain especially for calves, we proceeded as follows: (1) we studied the development of certain bacterial groups in healthy newborn calves, and (2) we investigated whether there are differences in terms of the quantitative development of these bacteria in healthy animals and animals that developed diarrhea (Schwaiger et al., 2020, 2022). Based on the study results, *L. reuteri* emerged as a possible diarrhea-preventive species.

As a first step, we verified the existing MALDI-TOF-MS identification results of 46 *L. reuteri* strains isolated from calf feces using species-specific PCR. Four isolates showed additional bands, which may be artifacts or due to contamination which led to their exclusion from further experiments. Nevertheless, this result again confirms that MALDI-TOF is a reliable identification method for bacteria (Dingle and Butler-Wu, 2013).

Further differentiation of the *L. reuteri* isolates using RAPD-PCR revealed 16 different band profile types, indicating gene variation within the species. However, it is striking that about one third of the *L. reuteri* isolates showed the same band profile, which might reflect that genetically distinct subpopulations of *L. reuteri* strongly correlate with their host and might indicate a strong host-symbiont association (Oh et al., 2010; Wegmann et al., 2015; Yu et al., 2018; Park et al., 2020).

Due to the risk of resistance transfer, EFSA guidelines specify that the susceptibility of a bacterial strain has to be tested for specific antibiotics if it is to be used as an additive in feed (EFSA Panel on Additives and Products or Substances used in Animal Feed, 2018). The goal is to minimize the spread of acquired resistance genes through horizontal gene transfer. At this point, it is important to point out again that we used doxycycline instead of tetracycline. Since doxycycline is approximately two to four times more potent than tetracycline (Dallas et al., 2013), this should be considered when evaluating MIC values. If the higher potency value of doxycycline is used as the basis for estimating a cut-off value, it would be set at 8 mg/L (compared to the EFSA cut-off value for tetracycline [32 mg/L]). If this estimate is used to evaluate the measured MIC values, 4 isolates show increased resistance to doxycycline. These isolates were excluded due to increased resistance to other antibiotics anyhow. The fact that only 2 of the 11 isolates tested met the criteria set indicates a quite significant prevalence of phenotypic antibiotic resistance in the *L. reuteri* species, a result comparable to that of Egervärn et al. (2007). Due to the growing problem of resistant bacteria in humans and animals, testing the antibiotic susceptibility of bacteria is an extremely important criterion when selecting candidates for a probiotic.

Further *in vitro* characterization of the two selected isolates (6-15-5Lac2 and 11-8-5Lac1) indicated that they could be suitable as potential probiotic microorganisms regarding the following aspects: (i) no exceptional cytotoxicity was observed in the MTT assay and the results were largely consistent with those of comparable studies (Motevaseli et al., 2013). At pH 6.5, both isolates showed even less cytotoxicity than the growth medium itself. The slightly higher cytotoxicity at low pH values of the culture medium is probably due to the acid produced by the test strains; it should be noted that the production of organic acids is considered a protective property of probiotic bacteria (O'Hara and Shanahan, 2007). According to the results of the study, together with the findings of other researchers, the selected isolates were classified as non-toxic, which supports the assumption that they are to be regarded as GRAS bacteria. (ii) *In vitro* assessment of the survivability of the two isolates in the gastrointestinal tract was also promising: both were found to be stable to high bile salt concentrations of up to 1.5%, which is approximately five times the physiological concentration in the chyme (Morelli, 2000). Only a pH of 2 resulted in a greater reduction in bacterial numbers within 4 h. Although the pH of the stomach contents of a fasting calf may fall below 2, it rises to values of 5 to 6 a few minutes after milk ingestion and then falls steadily to baseline within 6 to 8 h (Ahmed et al., 2002). Since both isolates were found to be stable at pH 3 and administration of the isolates was planned as part of the diet, there were no restrictions on their use as a possible probiotic.

The untreated culture supernatants (pH 4.4) of both *L. reuteri* isolates yielded low growth inhibition zones against the tested bacterial diarrheal pathogens (*E. coli* O0101:K99; *S. typhimurium C. perfringens*), but neutralization of the pH abolished the inhibitory effect. Since a growth medium adjusted to pH 4.4 did not result in the formation of an inhibition zone, the observed inhibitory effect cannot be attributed solely to the low pH. This is plausible since it is known that *L. reuteri* produces antimicrobial substances apart from lactic acid, such as ethanol, reuterin and reutericyclin (Mu et al., 2018). Additionally, it is also known that organic acids, such as lactic acid, exert their anti-bacterial effect in the acidic range (Ouwehand and Vesterlund, 2004). Since there were no investigations on the production of antimicrobial or otherwise protective substances in the present study, it would be interesting to have a closer look on those compounds in future research – ideally considering that, e.g., the gut microbiome and host immune responses can influence production and efficacy (Fernández et al., 2018; Fernández-Ciganda et al., 2022). When using organoid models, it may also be possible to make some statements about the mechanisms of action under near-realistic conditions, which could substantiate and explain the clinical effectiveness *in vitro* (Bozzetti and Senger, 2022).

A comparative four-day administration of the *L. reuteri* suspension or a placebo to newborn calves showed the diarrhea-protective potential of the two isolates: the incidence of diarrhea within the first 14 days was statistically significantly reduced by about 30%. This result may not seem very convincing at first sight, but it must be considered that the administration of the suspension was started only on the second day of life and that it lasted only 4 days. In addition, the experiments were conducted practically in 10 farms with different diarrhea incidences, which was taken into account in the chosen statistical model. It is well known that diarrhea in newborn calves occurs in two phases (about day 1 to 3 and about day 5 to 9; Lorino et al., 2005; Schwaiger et al., 2022). Since the first wave of diarrhea starts virtually after birth, its intensity might not have been influenced by administration of *L. reuteri* beginning on the second day; the delayed use of the *L. reuteri* suspension was due to a compromise with the farmers for logistical reasons. In addition, the growth kinetics of *L. reuteri* must also be considered. When culturing metabolically active *L. reuteri* strains in MRS broth (anaerobic, 37°C), the lag phase is between 2 and 5 h (Ehrmann et al., 2002). Our *L. reuteri* isolates were suspended in nutrient-free PBS which merely provides an environment to keep bacteria alive for a certain time, but it does not contain the nutrients which are necessary for the growth of *L. reuteri*. Moreover, they were stored at 4°C before administration, whereas optimum growth is around 37°C (DSMZ, 2023). Although it has not been verified in this study, it seems possible that under these suboptimal conditions their growth and metabolic activities are significantly reduced, which should result in a prolonged lag phase and thus a delayed onset of action.

Nevertheless, the incidence of diarrhea in the *L. reuteri* group was significantly lower than in the placebo group between days 4 and 10 of life. It can be assumed that administration of *L. reuteri* immediately after birth with the colostrum and over a longer period would have reduced the incidence of diarrhea even more significantly. The most effective administration regimen would need to be determined through an extensive follow-up study comparing different administration times and durations, where it would also be interesting to see to what extent other parameters such as weight gain, general health or, in the long term, subsequent fertility are influenced.

When looking at the incidence of known pathogens in the calves with diarrhea within the first week of life of both groups, no major differences can be observed. Only fewer rotavirus infections were detected in the *L. reuteri* group, compared to the placebo group (13% versus 28%), but this result is not statistically significant. However, this observation is not entirely uninteresting or to be dismissed out of hand, since inhibition of rotaviruses by *L. reuteri* has already been described in various studies (e.g., Alak et al., 1997; Sagheddu et al., 2020).

In summary, our studies confirm that *L. reuteri* are of particular importance for the intestinal health of newborn calves. The significant reduction of calf diarrhea in the first critical 14 days of life by a 4-day prophylactic administration of a mixture of *L. reuteri* 6-15-5Lac2 and 11-8-5Lac1 isolated from healthy calves impressively underlines this. The protective effect could be even more pronounced if an improved administration regimen is developed in terms of onset, frequency, and duration.

## Data availability statement

The datasets presented in this study can be found in online repositories. The names of the repository/repositories and accession number(s) can be found in the article/Supplementary material.

## Ethics statement

The requirement of ethical approval was waived by Government of Upper Bavaria (Germany) for the studies involving animals because the feeding experiment did not count as animal testing in the sense of the Animal Welfare Act due to the GRAS status of *L. reuteri*. The studies were conducted in accordance with the local legislation and institutional requirements. Written informed consent was obtained from the owners for the participation of their animals in this study.

## Author contributions

KS: Conceptualization, Funding acquisition, Methodology, Project administration, Supervision, Writing – review & editing. JS: Data curation, Formal analysis, Investigation, Methodology, Project administration, Validation, Writing – review & editing. CB: Data curation, Validation, Writing – review & editing. JB: Conceptualization, Formal analysis, Funding acquisition, Methodology, Project administration, Resources, Validation, Writing – original draft.

## Footnotes

^1^ http://blast.ncbi.nlm.nih.gov/Blast.cgi
