# Supplementary material for: Lactobacillus (Limosilactobacillus) reuteri: a probiotic candidate to reduce neonatal diarrhea in calves
Source: Front Microbiol. 2023 Oct 3;14:1266905. doi: 10.3389/fmicb.2023.1266905 (PMC10579909; doi:10.3389/fmicb.2023.1266905)
Supplement: Supplementary file 1 [file Table_1.DOCX]

Supplementary Table: Sequences of the two selected *L. reuteri* strains 11-8-5 Lac1 and 6-15-5 Lac2 determined by 16SrRNA-gene-PCR (primer pair: com1/com2; Korthals et al., 2008) and by species-specific PCR (primer pair: REUT1/LOWLAC; Chagnaud et al., 2001)

| Primer Pairs | Isolate | Sequence (5´- 3´) |
| --- | --- | --- |
| com1/com2 | 6-15-5 Lac 2 | CAGCAGCCGCGGTAATACGTAGGTGGCAAGCGTTATCCGGATTTATTGGGCGTAAAGCGAGCGCRGGCGGTTGCTTAGGTCTGATGTGAAAGCCTTCGGCTTAACCGAAGAAGTGCATCGGAAACCGGGCGACTTGAGTGCAGAAGAGGACAGTGGAACTCCATGTGTAGCGGTGGAATGCGTAGATATATGGAAGAACACCAGTGGCGAAGGCGRCTGTCTGGTCTGCAACTGACGCTGAGGCTCGAAAGCATGGGTAGCGAACAGGATTAGATACCCTGGTAGTCCATGCCGTAAACGATGAGTGCTAGGTGTTGGAGGGTTTCCGCCCTTCAGTGCCGGAGCTAACGCATTAAGCACTCCGCCTGGGGAGTACGACCGCAAGGTTGAAACTCAAAGGAATTGACGG |
|  | 11-8-5 Lac 1 | CAGCAGCCGCGGTAATACGTAGGTGGCAAGCGTTATCCGGATTTATTGGGCGTAAAGCGAGCGCAGGCGGTTGCTTAGGTCTGATGTGAAAGCCTTCGGCTTAACCGAAGAAGTGCATCGGAAACCGGGCGACTTGAGTGCAGAAGAGGACAGTGGAACTCCATGTGTAGCGGTGGAATGCGTAGATATATGGAAGAACACCAGTGGCGAAGGCGRCTGTCTGGTCTGCAACTGACGCTGAGGCTCGAAAGCATGGGTAGCGAACAGGATTAGATACCCTGGTAGTCCATGCCGTAAACGATGAGTGCTAGGTGTTGGAGGGTTTCCRCCCTTCAGTGCCGGAGCTAACGCATTAAGCACTCCGCCTGGGGAGTACGACCGCAAGGTTGAAACTCAAAGGAATTGACGG |
| REUT1/ LOWLAC | 6-15-5 Lac 2 | CGACGACCATGAACCACCTGTCATTGCGTCCCCGAAGGGAACGCCTTATCTCTAAGGTTAGCGCAAGATGTCAAGACCTGGTAAGGTTCTTCGCGTAGCTTCGAATTAAACCACATGCTCCACCGCTTGTGCGGGCCCCCGTCAATTCCTTTGAGTTTCAACCTTGCGGTCGTACTCCCCAGGCGGAGTGCTTAATGCGTTAGCTCCGGCACTGAAGGGCGGAAACCCTCCAACACCTAGCACTCATCGTTTACGGCATGGACTACCAGGGTATCTAATCCTGTTCGCTACCCATGCTTTCGAGCCTCAGCGTCAGTTGCAGACCAGACAGyCGCCTTCGCCACTGGTGTTCTTCCATATATCTACGCATTCCACCGCTACACATGGAGTTCCACTGTCCTCTTCTGCACTCAAGTCGCCCGGTTTCCGATGCACTTCTTCGGTTAAGCCGAAGGCTTTCACATCAGACCTAAGCAACCGCCYGCGCTCGCTTTACGCCCAATAAATCCGGATAACGCTTGCCACCTACGTATTACCGCGGCTGCTGGCACGTAGTTAGCCGTGACTTTCTGGTTGGATACCGTCACTGCGTGAACAGTTACTCTCACGCAYRTTCTTCTCCAACAACAGAGCTTTACGAGCCGAAACCCTTCTTCACTCACGCGGTGTTGCTCCATCAGGCTTGCGCCCATTGTGGAAGATTCCCTACTGCTGCCTCCCGTAGGAGTATGGACCGTGTCTCAGTTCCATTGTGGCCGATCAGTCTCTCAACTCGGCTATGCATCATYGCCTTGGTAAGCCGTTACCTTACCAACTAGCTAATGCACCGCAGGTCCATCCCAGAGTGATAGCCAAAGCCATCTTTCAAAYAAAARCCATGYGGCTTTTRTTGTTATGCGGTATTAGCATCTGTTTCCAAATGTTATCCCCCGCTCCGGGGCRGGTTACCTACGTGTTACTCACCCGTCCGCCACTCACTGGTGATCCATCGTCAATTCA |
|  | 11-8-5 Lac 1 | CGACGACCATGAACCACCTGTCATTGCGTCCCCGAAGGGAACGCCTTATCTCTAAGGTTAGCGCAAGATGTCAAGACCTGGTAAGGTTCTTCGCGTAGCTTCGAATTAAACCACATGCTCCACCGCTTGTGCGGGCCCCCGTCAATTCCTTTGAGTTTCAACCTTGCGGTCGTACTCCCCAGGCGGAGTGCTTAATGCGTTAGCTCCGGCACTGAAGGGYGGAAACCCTCCAACACCTAGCACTCATCGTTTACGGCATGGACTACCAGGGTATCTAATCCTGTTCGCTACCCATGCTTTCGAGCCTCAGCGTCAGTTGCAGACCAGACAGyCGCCTTCGCCACTGGTGTTCTTCCATATATCTACGCATTCCACCGCTACACATGGAGTTCCACTGTCCTCTTCTGCACTCAAGTCGCCCGGTTTCCGATGCACTTCTTCGGTTAAGCCGAAGGCTTTCACATCAGACCTAAGCAACCGCCTGCGCTCGCTTTACGCCCAATAAATCCGGATAACGCTTGCCACCTACGTATTACCGCGGCTGCTGGCACGTAGTTAGCCGTGACTTTCTGGTTGGATACCGTCACTGCGTGAACAGTTACTCTCACGCACGTTCTTCTCCAACAACAGAGCTTTACGAGCCGAAACCCTTCTTCACTCACGCGGTGTTGCTCCATCAGGCTTGCGCCCATTGTGGAAGATTCCCTACTGCTGCCTCCCGTAGGAGTATGGACCGTGTCTCAGTTCCATTGTGGCCGATCAGTCTCTCAACTCGGCTATGCATCATYGCCTTGGTAAGCCRTTACCTTACCAACTAGCTAATGCACCGCAGGTCCATCCCAGAGTGATAGCCAAAGCCATCTTTCAAACAAAARCCATGYGGCTTTTRTTGTTATGCGGTATTAKCATCTGTTTCCAAATGTTATCCCCCGCTCCGGGGCAGGTTACCTACGTGTTACTCACCCGTCCGCCACTCACTGGTGATCCATCGTCAATTCA |
